# Green-Fluorescent Protein^+^ Astrocytes Attach to Beta-Amyloid Plaques in an Alzheimer Mouse Model and Are Sensitive for Clasmatodendrosis

**DOI:** 10.3389/fnagi.2016.00075

**Published:** 2016-04-08

**Authors:** Nina Daschil, Christian Humpel

**Affiliations:** Laboratory of Psychiatry and Experimental Alzheimer’s Research, Department of Psychiatry, Psychosomatics and Psychotherapy, Medical University of InnsbruckInnsbruck, Austria

**Keywords:** astrocytes, Alzheimer’s disease, GFAP, GFP, beta-amyloid, clasmatodendrosis

## Abstract

Alzheimer’s disease (AD) is pathologically characterized by beta-amyloid (Aβ) plaques and Tau pathology. It is well-established that Aβ plaques are surrounded by reactive astrocytes, highly expressing glial fibrillary acidic protein (GFAP). In order to study the cellular interaction of reactive astrocytes with Aβ plaques, we crossbred mice overexpressing amyloid precursor protein (APP) with the Swedish-Dutch-Iowa mutations (APP-SweDI) with mice expressing green fluorescent protein (GFP) under the GFAP-promotor. Three-dimensional confocal microscopy revealed a tight association and intense sprouting of astrocytic finely branched processes towards Aβ plaques in 12 month old mice. In order to study phagocytosis, 110 μm thick brain slices from 12 month old crossbred mice were cultured overnight, however, we found that the GFP fluorescence faded, distal processes degenerated and a complete loss of astrocytic morphology was seen (clasmatodendrosis). In summary, our data show that GFP^+^ reactive astrocytes make intense contact with Aβ plaques but these cells are highly vulnerable for degeneration.

## Introduction

Alzheimer’s disease (AD) comes along with a severe cognitive decline and is characterized by the extracellular deposition of beta-amyloid (Aβ) plaques, intraneuronal Tau pathology, synaptic loss resulting in cholinergic neuronal cell death and inflammatory processes such as the activation of astrocytes and microglia (Selkoe, 2001). It is well-established that Aβ plaques in AD are surrounded by reactive gial-fibrillary acidic protein (GFAP)^+^ astrocytes (Sofroniew and Vinters, 2010; Colangelo et al., 2014; Pekny et al., 2014). Recent studies suggest also beneficial roles for astrogliosis in AD. In fact, reactive astrocytes surrounding plaques can release matrix-metalloproteinases or neprilysin causing degradation of Aβ plaques (Lim et al., 2011; Yan et al., 2006; Webster et al., 2014). Moreover, there is evidence that reactive astrocytes can also phagocyte Aβ plaques, as shown by incorporating FITC-labeled Aβ peptides (Wyss-Coray et al., 2003; Jones et al., 2013). However, so far to our best knowledge, a detailed 3-Dimensional confocal imaging of the cellular interaction between reactive astroglia and plaques has not yet been shown.

We recently reported that in an AD mouse model reactive astrocytes in proximity to plaques express a calcium channel subunit, as well as neuropeptides, possibly playing a role in angiogenic responses (Daschil et al., 2013, 2015). To gain more cellular insight into the role of reactive astrocytes surrounding Aβ plaques we cross-bred the same transgenic AD mice with mice expressing green fluorescent protein (GFP) under the GFAP-promotor. By means of immunohistochemistry and 3D confocal microscopy we aimed to characterize these GFP^+^ reactive astrocytes surrounding Aβ plaques in the cortex. Further, we aimed to culture brain slices of these mice to follow up the morphology of these GFP^+^ cells, especially to test for phagocytosis.

## Materials and Methods

Transgenic Alzheimer mice were purchased from MMRRC (USA). These animals express the human amyloid precursor protein (APP) harboring the Swedish K670N/M671L, Dutch E693Q and Iowa D694N mutations and have been extensively characterized (Davis et al., 2004). Generation of APP-SweDI × GFP-GFAP mice was established by crossing the animals previously mentioned to mice expressing enhanced GFP (eGFP) under the promotor of GFAP (Nolte et al., 2001; kindly provided by Prof. Kettenmann, Berlin). All experiments were performed in accordance to Austrian animal protection and welfare act.

Twelve month old mice were anesthetized (Ketamin 100 mg/kg/ Xylazine 10 mg/kg) and subsequently transcardially transcardially perfused with phosphate-buffered saline and 4% paraformaldehyde immersed in 20% sucrose. Brains were frozen in a CO_2_ stream and cut into coronal sections of 40 μm with a cryostat. Immunohistochemistry was performed as described in detail (Daschil et al., 2013, 2015). After pretreatment, sections were incubated with primary antibodies (Aβ, 1:1000, Sigma A8978; GFAP, 1:2000, Millipore AB5541) for 2–3 days at 4°C. Sections were rinsed and incubated with the secondary antibody (1:400; Alexa Fluor 546, Invitrogen). Fluorescent sections were analyzed using an Olympus BX61 (ProgRes C14 camera) microscope equipped with an OpenLab 5.5.0 imaging software. Z-stacks were recorded by using a SP5 confocal microscope (Leica Microsystems, Wetzlar, Germany) with a HCX PL APO 63×/1.3 NA glycerol objective. Imaging of eGFP and Alexa 546 was performed using an argon laser and a DPSS561 laser, respectively. Emission of fluorophores was detected from 493 to 556 nm (eGFP) and 566 to 628 nm (Alexa546). Images were acquired by using the LAS AF acquisition software (Version 2.1.), further deconvoluted with the Huygens Professional software (Scientific Volume Imaging, Netherlands) and finalized with the Imaris 8 software (Bitplane AG, Switzerland).

To study cultured GFP^+^ astrocytes some brains were sectioned using a cooled vibratome as described in detail for organotypic brain slices (Ullrich et al., 2011). The animals were sacrificed and the brains were dissected. Vibrosections (110 μm) were sagittally cut and incubated in slice medium overnight at 37°C, 5% CO_2_. Live cell imaging was performed with a Leica DM IRB inverse microscope and photos were taken after 0–6 h of incubation.

Western blot analysis was carried out as described previously (Hochstrasser et al., 2011). Brain extracts were loaded onto polyacrylamide gels and electrophoresis was performed for 35 min at 200 V. Protein was subsequently electrotransferred to nylon PVDF Immobilon-PSQ membranes. For detection, the Western Breeze Chemiluminescent System (Invitrogen) was used. Blots were incubated with the primary anti-eGFP (1:1000, Abcam ab184601), anti-GFAP (1:2000, Millipore, AB5541) or anti-actin (1:500; Sigma, A2066) antibodies.

## Results

### Association of GFP^+^ Reactive Astrocytes with Aβ Plaques

**Figure 1:**
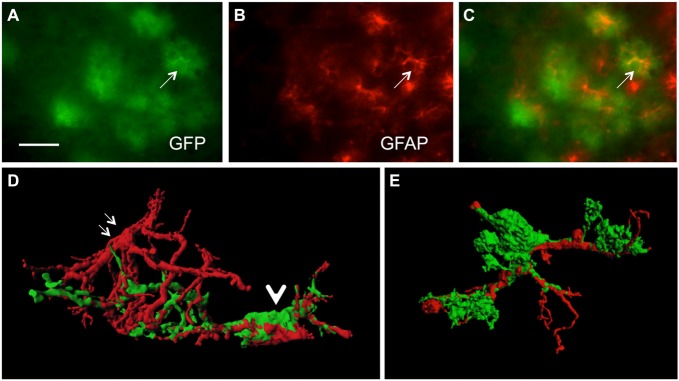
**Colocalization of green fluorescent protein (GFP)^+^ and glial fibrillary acidic protein (GFAP)^+^ astrocytes.** Sections of amyloid precursor protein (APP)-Swedish-Dutch-Iowa (SweDI) × GFP-GFAP mice were immunohistochemically stained for GFAP **(B–E)**. In a cluster of reactive astroglia, many cells were double positive for both GFP (green, **A**) and GFAP (red, indicated by an arrow, **A–C**). However, some astrocytes were positive for either GFP or GFAP **(A–C)**. Confocal microscopy of brain sections clearly shows double positive astrocytes (indicated by a thick arrow **D,E**) adjacent to an astrocyte being merely positive for GFAP (indicated by thin arrows, **D**). Scale bar = 35 μm **(A–C)**, 6 μm **(D,E)**.

**Figure 2:**
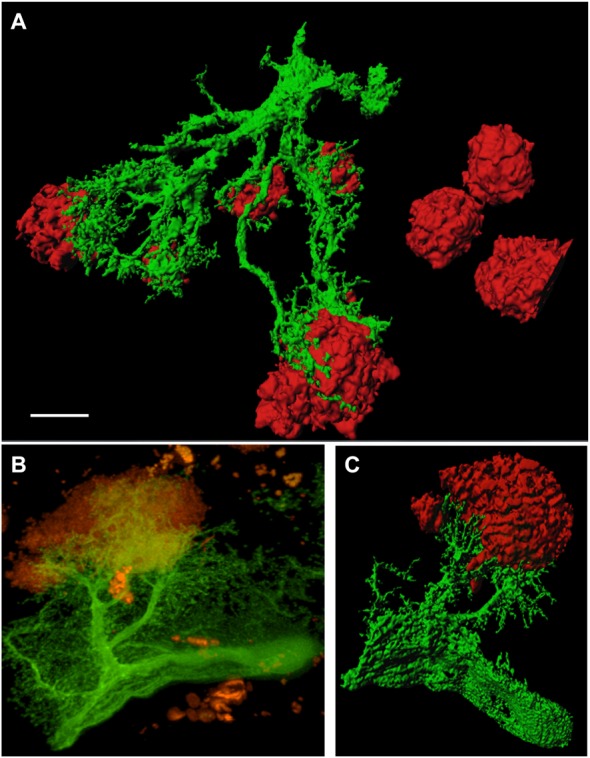
**GFP^+^ astrocytes extend towards beta-amyloid (Aβ) plaques in 12 month old APP-SweDI × GFP-GFAP mice.** 3D-imaging reveals the tight association of reactive astrocytes (green) with Aβ plaques (red; **A,C**). Thick astrocytic branches extend towards Aβ plaques and fine processes showed intense arborization **(B)**. Scale bar = 5 μm **(A)**, 7 μm **(B,C)**.

In order to study GFP^+^ reactive astrocytes surrounding Aβ plaques we established a new mouse model (the crossbred APP-SweDI × GFP-GFAP mice). As expected, the Aβ plaque density was very high in 12 month old mice in the cortex, amygdala, thalamic nuclei and to a lesser extent in hippocampi (data not shown). GFP^+^ astrocytes were expressed predominantly in cortical and thalamic areas (data not shown). However, approx. 30–45% of all animals did not show GFP^+^ cells. The GFP^+^ astrocytes were highly positive for GFAP (Figures 1A–C) but also several GFP negative astrocytes were observed (Figures 1A–C). 3D Confocal microscopy confirmed the colocalization of GFP^+^ and GFAP^+^ astrocytes (Figures 1D,E). Interestingly, GFAP was predominantly distributed in the main branches and perinuclear region of astrocytes whereas GFP was additionally expressed in fine ramified processes (Figures 1D,E). Some double positive astrocytes were surrounded by single GFAP^+^ astroglia (Figure 1D). By means of confocal microscopy and subsequent 3D-imaging, we could demonstrate a tight association between reactive GFP^+^ astrocytes and some Aβ plaques. Interestingly, thick branches of astrocytes directly extended towards Aβ deposits (Figures 2A–C). An extensive spreading of numerous thin and ramified processes was observed in all directions but particularly towards Aβ plaques (Figure 2B).

### Morphology of GFP^+^ Astrocytes in Culture

**Figure 3:**
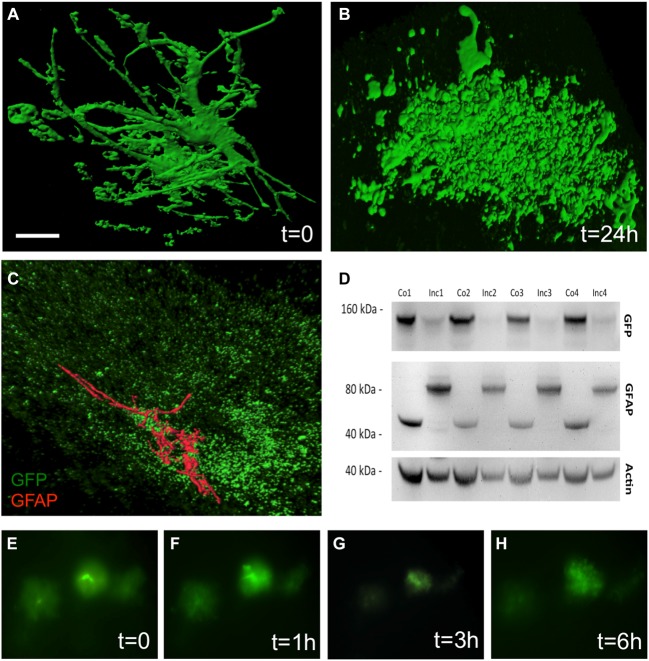
**Clasmatodendrosis in cultured astrocytes of 12 month old APP-SweDI × GFP-GFAP mice.** Healthy astrocytes were detected in 110 μm vibratome brain slices immediately fixed after sectioning. Note the distinct processes and arborization **(A)**. However, 24 h after culturing cells dramatically changed their morphology resulting in the loss of distal processes as well as the generation of isolated fluorescent bodies with no connection to the former cell **(B)**. Some clasmatodendritic cells still showed the presence of a few slight glial fibrilllary acidic protein (GFAP)^+^ branches **(C)**. Western blot analysis revealed a decrease in GFP, whereas the GFAP protein was enhanced in size after 24 h of culturing **(D)**. Size marker are given in kDa. *In vitro* live cell imaging shows that many GFP^+^ cells degenerate already within 3 h of culturing **(E–H)**. Scale bar = 5 μm **(A,B)**, 7 μm **(C)**, 40 μm **(E–H)**.

The culturing of vibratome brain slices over 24 h caused a dramatic change in astrocytic shape, a process which is termed “clasmatodendrosis” (Figure 3B). In comparison, those brain slices which were not cultured overnight showed healthy GFP^+^ astrocytes with intense arborization (Figure 3A). Immunohistochemistry revealed the presence of GFAP in a few GFP^+^ astrocytes affected by clasmatodendrosis (Figure 3C). GFP^+^ astrocytes almost completely and rapidly lost fluorescence during culturing as seen in experiments using live cell imaging (Figures 3E–H). This fading of GFP fluorescence was also observed by means of Western blot analysis, clearly showing a strong decrease of GFP protein after an incubation period of 1 day compared to control protein levels analyzed on day 0 (Figure 3D). Additionally, we observed an increase in the molecular weight of GFAP (from 40 to 80 kDa) after 24 h of incubation suggesting an aggregation of this intermediate filament protein (Figure 3D).

## Discussion

By employing confocal microscopy together with 3D imaging we reveal a close interaction between reactive astrocytes and Aβ plaques. In this study we could show that reactive astroglia extend their processes towards extracellular deposits of Aβ protein in the brain of transgenic AD mice. Further analyses demonstrate that prolonged *in-vitro* culture of brain slices results in clasmatodendrosis of reactive astrocytes.

### Reactive GFP^+^ Astrocytes Surrounding Aβ Plaques

It is well-established that Aβ plaques are surrounded by reactive GFAP^+^ astrocytes in AD mouse brains (Nagele et al., 2004; Olabarria et al., 2010; Daschil et al., 2013, 2015; Serrano-Pozo et al., 2013; Rodríguez-Arellano et al., 2015). In order to study the morphology of reactive astrocytes in proximity to Aβ plaques we cross-bred APP-SweDI mice (Davis et al., 2004) with GFP-GFAP mice (Nolte et al., 2001), harboring GFP in astrocytes. Reminiscent to previous studies (Nolte et al., 2001) 30–45% of the mouse offspring did not harbour GFP^+^ astrocytes, nevertheless all littermates were fully viable. The reason for the loss of GFP in these crossbred mice is not known, however, an active metabolism and turnover of GFP and loss of fluorescence, an enhanced GFP toxicity or an increased sensitivity of GFP^+^ astrocytes for cell death may occur. We could show that all animals exhibited severe plaque load in 12 month old mice as shown previously (Daschil et al., 2013). Interestingly, GFP^+^ astrocytes also appeared in areas with less or even no Aβ plaques, which is in line with Simpson et al. (2010). In accordance with Nolte et al. (2001) GFP did not completely overlap with GFAP^+^ astrocytes. This is due to the different expression patterns of these proteins, since GFP is expressed in the cytoplasm and fine processes. On the other hand, GFAP is mainly localized in cytoskeletal perinuclear domains and thick processes arranged in intermediate filament bundles (Nolte et al., 2001; Suzuki et al., 2003).

### GFP^+^ Reactive Astrocytes Make Contact with Aβ Plaques

Several studies have proven an association and sometimes even a penetration of reactive astrocytes with Aβ plaques (Serrano-Pozo et al., 2013). In the majority of cases, astroglia were visualized by an antibody directed against GFAP since this is the most appropriate marker to detect reactive astrocytes (Sofroniew and Vinters, 2010). However, we could show now for the first time a clear extension of thick and particularly finely branched astrocytic processes which were GFP positive and directed towards Aβ plaques by means of 3D confocal microscopy. In addition, we could nicely demonstrate that the astrocytic processes not just penetrated but also clasped around Aβ deposits. Since GFAP expression is predominantly restricted to perinuclear domains and main branches, this marker is not suitable to visualize the finely arborized processes of astroglia.

### Culturing of GFP^+^ Astrocytes and Clasmatodendrosis

It has been shown that reactive astrocytes are capable of phagocytosing Aβ deposits or dead cells after brain injury and thereby protecting surrounding healthy neurons from cell death (Wyss-Coray et al., 2003; Lööv et al., 2012; Jones et al., 2013). The occurrence of lysosomes additionally strengthens the hypothesis that astrocytes are involved in phagocytosis (Jones et al., 2013). In order to study phagocytosis of GFP^+^ astrocytes, we cultured brain sections of 12 month old mice overnight. However, we observed a gradual degradation of astrocytic morphology and a loss of GFP fluorescence upon prolonged *in vitro* culturing already 3 h after dissection of cortical brain slices. For that reason it was difficult to perform extensive analysis of phagocytosis by astrocytes. We found that GFP^+^ astrocytes entirely lost their distal processes giving rise to isolated fluorescent bodies resulting in a process termed clasmatodendrosis. There are clear indications that astrocytes undergo clasmatodendrosis after hyperglycemia or ischemia or acidosis (Duchen, 1992; Hulse et al., 2001) but also in AD (Tomimoto et al., 1997; Sahlas et al., 2002; Mercatelli et al., 2016). Our data show that GFP^+^ astrocytes undergo clasmatodendrosis already within 3 h, which was surprising, because we used a well-defined serum medium and conditions which are neither acidic nor ischemic. Such a medium has been proven to be suitable to culture astroglia in organotypic brain slices for several weeks. Thus, it can be concluded that these GFP^+^ astrocytes are very sensitive for cell death.

Taken together, our data show for the first time a detailed cellular 3D confocal imaging of reactive astrocytes around plaques, showing small ramified processes towards Aβ plaques. By culturing organotypic brain slices we could observe a severe change in morphology such as the complete loss of astrocytic processes as well as the generation of isolated fluorescent bodies. In conclusion, this study provides insights into the cellular interaction of activated astrocytes in relation with plaques, further supporting the phagocytic role of reactive astrocytes in AD.

## Author Contributions

ND performed all experiments and contributed to writing the article. CH designed the experiments and wrote the article.

## Funding

This study was supported by the Austrian Science Fund (F4405-B19 and P24541-B24).

## Conflict of Interest Statement

The authors declare that the research was conducted in the absence of any commercial or financial relationships that could be construed as a potential conflict of interest.

## Abbreviations

Aβ: Beta-amyloid
AD: Alzheimer’s disease
APP: Amyloid precursor protein
GFAP: Glial fibrillary acidic protein
GFP: Green fluorescent protein.
